# Longitudinal Meta-Analysis of Peak Height Velocity in Young Female Athletes

**DOI:** 10.7759/cureus.59482

**Published:** 2024-05-01

**Authors:** Ahlan B Lima, Ricardo T Quinaud, Fábio C Karasiak, Luciano G Galvão, Carlos E Gonçalves, Humberto M Carvalho

**Affiliations:** 1 School of Sports, Federal University of Santa Catarina, Florianópolis, Santa Catarina, BRA; 2 Department of Physical Education, University of the Extreme South of Santa Catarina, Criciúma, BRA; 3 Faculty of Sport Sciences and Physical Education, University of Coimbra, Coimbra, PRT

**Keywords:** longitudinal studies, multilevel analysis, bayesian approach, adolescent development, youth sports

## Abstract

Growth patterns and biological milestones in youth sports are key to interpreting the development of young athletes. However, there is no analysis of longitudinal meta-analysis describing the growth of young female athletes. This longitudinal meta-analysis estimated growth curves and age at peak height velocity (PHV) in young female athletes based on anthropometric data from longitudinal studies found in the literature. Following the Preferred Reporting Items for Systematic Reviews and Meta-Analyses (PRISMA) guidelines, studies with repeated measurements in young female athletes were identified from searches of four databases (MEDLINE, Web of Science, SCOPUS, and SPORTDiscus) without date restrictions through August 2023. We adapted our bias assessment criteria using the Cochrane risk of bias tool for randomized controlled trials as a reference. Bayesian multilevel modeling was used to perform a longitudinal meta-analysis to extract stature growth curves and age at PHV. Fourteen studies met our eligibility criteria. Twenty-one independent samples could be included in the analysis. Conditional on the data and models, the predicted mean age at PHV for female athletes was 11.18 years (90% CI: 8.62; 12.94). When studies were aggregated by sport in the models, the models could not capture sport-specific growth curves for stature and estimate a corresponding age at PHV. We provide the first longitudinal meta-analytic summary of pubertal growth and derive age at PHV in young female athletes. The meta-analysis predicted that age at PHV occurs at similar ages to those in the general pediatric population. The data pool was limited in sports and geographic distribution, emphasizing the need to promote longitudinal research in females across different youth sports contexts.

## Introduction and background

The advance in our understanding of young athletes' growth and development can only be studied with repeated measures of individual athletes over a period of time [1]. Biological maturation milestones and pubertal growth patterns, including peak height velocity (PHV) during puberty and age at PHV, require longitudinal measurements [1,2]. Adding to the value of longitudinal data in youth sports, the synthesis of this data would provide meaningful insights for coaches, researchers, and relevant stakeholders. For example, variations in the patterns of growth of players pooled by sport may provide deeper insights into the trends of selection in youth sports. Recently, we used a longitudinal meta-analysis [3] to describe pubertal growth patterns of stature in young male athletes. Data were extracted from 30 longitudinal studies with appropriate designs, allowing us to describe the growth and maturation of male athletes during adolescence. Although the available data are limited (e.g., data pool limitations or lack of consistent and transparent reporting), it was possible to note gaps in longitudinal research in most sports other than football from European countries.

Female athletes are gaining more attention in recent years due to their participation and performance in major sporting events [4]. Despite this, female athletes remain underrepresented in sports science and sports medicine research, particularly in youth sports [5]. Furthermore, training and performance guidelines have been based primarily on male data, with insufficient attention paid to female athletes [6,7]. This disparity in research leads to various gaps in our knowledge of the development of young female athletes and challenges coaches and stakeholders to establish evidence-based practices [8]. In particular, the knowledge about the variation in growth patterns and biological milestones in young female athletes is limited, at best. Hence, the description of the age at which PHV occurs based on available data can provide specific information to interpret young female athletes’ development and performance.

Adolescence is a time marked by several changes in body shape, size, composition, physical abilities, and behavior. In particular, growth and maturation are influential processes that significantly affect the athletic performance of young female athletes and their participation in sports. It is therefore essential to have reference information on changes in body dimensions during pubertal growth in young female athletes [9].

Meta-analyses of longitudinal data provide an efficient method to objectively describe development patterns in young athletes by summarizing the existing evidence on a research topic. Our study estimated growth curves and age at PHV in young female athletes based on anthropometric data from longitudinal studies found in the literature. To conduct the longitudinal meta-analysis, we used Bayesian multilevel modeling and fitted polynomial models.

## Review

Methods

Search Strategy

The study protocol was registered in the International Prospective Register of Systematic Reviews - PROSPERO (registration number: CRD42020175084). Our data synthesis was carried out and presented following the Preferred Reporting Items for Systematic Reviews and Meta-Analysis (PRISMA and PRISMA-P) guidelines [10,11]. Our methodology was adapted to identify longitudinal studies reporting repeated measures of stature in young female athletes, following a previous study in young male athletes [3].

Selection Criteria

We conducted an electronic search from the inception of relevant databases to August 2, 2023, across four databases: MEDLINE (via PubMed, 1946 to present), SCOPUS (Elsevier), Web of Science (Core Collection, 1945 to present), and SPORTDiscus (via EBSCOhost).

Our search strategy carefully identified longitudinal studies that measured stature in young athletes. Eligible articles had to be published in peer-reviewed journals. Articles that met the specified criteria had to be written in English and published in peer-reviewed journals. There were no publication date restrictions. Our search strategy adhered to the PICOS framework (Patients, Intervention, Comparator, Outcomes, and Study Type). However, we did not include the comparator element which was not applicable in this study since no group comparisons were involved. Search terms were organized into distinct clusters, as follows: (1) Population (e.g., “athlete,” “player”); (2) Population age (e.g., “youth,” “adolescent”); (3) Intervention (e.g., “sports,” “soccer,” “basketball”); (4) Outcomes (e.g., “height,” “stature”); and (5) Study type (e.g., “longitudinal,” “repeated measures”).

For complete coverage, our search was conducted in two stages. Firstly, we searched for relevant articles within each cluster using the Boolean operator ‘OR’ to combine terms within a cluster. Secondly, we used the Boolean operator ‘AND’ to link the clusters. Our preliminary search for keywords within electronic databases considered ‘All fields’, except for SCOPUS where we applied the TITLE-ABS-KEY (title, abstract, and keywords) filter. However, in subsequent search updates across all databases, we implemented the “title, abstract, and keywords” filter primarily due to the many articles retrieved. For a comprehensive description of our search strategy, see the supplementary materials (https://osf.io/znrck/).

Study Inclusion and Exclusion Criteria

For inclusion in our meta-analysis, studies had to meet the following criteria: (1) Longitudinal Design - studies were required to have a longitudinal design, tracking individuals over a minimum duration of six months; (2) repeated measures - studies needed to report a minimum of three repeated measures for stature between the ages of 8 and 15 years; (3) sample of young female athletes - the study should have involved a sample exclusively composed of young female athletes; and (4) original articles in English - only original articles written in the English language and published in peer-reviewed journals were considered.

Conversely, studies were excluded from the meta-analysis if they met any of the following criteria: (1) mixed samples - studies that included a mixed sample and did not report data separately by sex were excluded; (2) incomplete reporting - case studies or conference proceedings with incomplete reporting were not included; (3) data reporting issues - studies that did not provide data suitable for analysis (e.g., lack of sample information by assessment points or chronological age) were excluded; and (4) incomplete description of training environment - studies lacking a comprehensive description of the training and competition environment of young female athletes were also excluded.

Article Selection Process

Two authors (ABL and RTQ) independently performed the article selection process based on the eligibility criteria. In the initial screening, they reviewed the titles and abstracts, selecting studies that satisfied the eligibility criteria or lacked clear aims and methods based on the abstracts. After obtaining full-text versions of the remaining studies, the authors independently applied the selection criteria. Studies meeting the inclusion criteria were included in the data synthesis for the meta-analysis.

Where there were disagreements between the two reviewers, these were resolved through discussion. Where necessary, a third reviewer (HMC) was involved. We used the Rayyan website [12] to facilitate the peer-review screening process and to ensure adherence to the eligibility criteria for titles, abstracts, and full-text assessments.

Data Extraction

We used the *WebPlotDigitizer* tool to extract data, mainly when information was presented exclusively in graphical plots or figures. In cases where a single large study produced different articles, we used our judgment to select the article that described and presented the results most comprehensively and accurately. We excluded all other articles from the analysis to avoid duplication. Our examination of potential duplicate data considered various factors, such as the list of authors, corresponding authors, research project identification, and data description within corresponding reports. This thorough assessment allowed us to make informed decisions regarding the inclusion of specific articles and ensured the avoidance of data duplication, ultimately enhancing the robustness and accuracy of our meta-analysis.

Assessment of Bias and Publication Bias in Repeated-Measures Meta-Analysis

Current guidelines for assessing the risk of publication bias in meta-analyses predominantly consider randomized clinical trials [13] or non-randomized clinical trials [14]. However, our meta-analysis focuses on longitudinal observational studies that report repeated measures of stature throughout adolescence among young athletes. Considering this context, we adapted our bias assessment criteria, taking the Cochrane risk of bias tool designed for randomized controlled trials [13] as a reference, but tailoring the criteria to align with our meta-analysis objectives, the targeted outcome, and the characteristics inherent to repeated measures designs.

Our bias assessment centered on four key domains [3]: (1) study design - evaluating the suitability of the study design in addressing our research question; (2) incomplete data or attrition - scrutinizing the handling of incomplete data or attrition within the selected studies; (3) measurement of the outcome - assessing the precision and reliability of the outcome measurements; and (4) selective reporting - investigating the potential for selective reporting of results. To adapt these domains to the specifics of our meta-analysis research question, we formulated adjusted signaling questions for each domain following our previous study with longitudinal meta-analysis [3].

The assessment of funnel plot asymmetry for longitudinal meta-analyses of continuous outcomes from observational studies is limited. Traditional methods for identifying publication bias [15] have raised concerns when applied to meta-analyses involving continuous outcomes [16]. We adopted a two-pronged approach to evaluate funnel plot asymmetry: (1) Egger regression test - employing the conventional Egger regression test [15] as a diagnostic tool for publication bias detection; (2) alternative approach - utilizing an alternative method that leverages meta-analysis model regression residuals in conjunction with the study's sample size [16] to assess funnel plot asymmetry. Detailed codes for the models used in the bias assessment are available at https://osf.io/znrck/.

Statistical Analysis

Model specification: We fitted a longitudinal meta-analysis model to describe the growth patterns of young female athletes, using a fully Bayesian approach [3]. Our model starts from a basic Bayesian meta-analysis model as follows:

$y_j \sim Normal(\theta_j,\sigma_j= se_j)$

where $y_j$ is the point estimate for the stature of a single study $j$, drawn from a normal distribution that is centered on that study's ``true'' estimate, $\theta_j$, and with a standard deviation $\sigma_j$ given by the study's standard error $se_j$. These are taken directly from each study $j$. We assumed that each study $j$ is drawn from a population of related studies, each with its point estimate for stature and respective standard error. Hence, it is assumed that $\theta_j$ has a normal distribution, where $\mu_j$ is the population mean (meta-analytic) estimate, and $\tau$ is the variation around the population mean, i.e., between-study variation:

$\theta_j \sim Normal(\mu_j,\tau)$

Following our previous modeling of longitudinal data studies [3], we specified the longitudinal meta-analysis model describing the growth patterns of young female athletes by considering each study's outcome $y_j$, i.e., stature, at time point $t$ (i.e., chronological age) in study $j$, with an error variance of the within-study sampling errors, assumed to be normally distributed. We fitted a nonlinear multilevel model (polynomial) on each study's maximum stature velocity of growth at puberty, considering at least growth coefficients up to the cubic to capture the known shape of pubertal growth. We choose our model given: the age range between 8 and 15 years, corresponding to a known shape of pubertal growth (sigmoidal); the flexibility to allow for group-level variation at the intercept and the slopes (between-study variation in the parameters), and regularized the estimates both with the available knowledge and each study data; fitting models with high complexity and Bayesian methods raises severe computational demands. As noted in our previous work [3], nonlinear multilevel models are often conceptually and computationally inconvenient in frequentist contexts [17], but are conceptually simple and computationally tractable in Bayesian frameworks [18].

In the general form of our model,$\mu_j$ is given by the intercept $\alpha_{j[t]}^{age}$, the linear, $\beta_{j[t]}^{age}$, and nonlinear terms,$\beta_{j[t]}^{age^2}$ and $\beta_{j}^{age^3}$. The notation for the $\mu_j$, $\beta_{j[t]}^{age}$, and $\beta_{j[t]}^{age^2}$ indicates that each study $j$ is given a unique intercept, linear and the second-order slopes are issued from a normal distribution centered, respectively on $\mu$, $\beta^{age}$, and $\beta^{age^2}$, the grand intercept and slopes, meaning that there might be different mean scores for each study. The correlation between the varying intercepts and the varying slopes was estimated by modeling $\alpha_{j[t]}^{age}$, $\beta_{j[t]}^{age}$, and $\beta_{j[t]}^{age^2}$ as issued from the same multivariate normal distribution (a multivariate normal distribution is a generalization of the usual normal distribution to more than one dimension), centered on 0 and with some covariance matrix S, as specified in the following form:

$\mu_j = \alpha_{j[t]}^{age} + \beta_{j[t]}^{age}+ \beta_{j[t]}^{age^2} + \beta_{j}^{age^3}$

\begin{bmatrix} \alpha_{j[t]}^{age} \\ \beta_{j[t]}^{age} \\ \beta_{j[t]}^{age^2} \\ \end{bmatrix} \sim MVNormal \begin{pmatrix} \begin{bmatrix} 0\\ 0\\ 0 \end{bmatrix}, S \end{pmatrix}\\

For computational efficiency, we set the correlation between intercepts and slopes used for the computation of the covariance matrix to 0. Also, we centered the outcomes on the aggregated sample means (sample mean for stature = 153.06 cm; sample mean for age = 12.43 years).

We also extended our model to predict age at PHV by sports. We aggregated the data by sport, allowing for the intercept and slopes (linear and second-order parameters) to vary by studies aggregated by sport. To adjust for each study variation across all population-level parameters, we allowed the intercept to vary by study. There was no significant variation by study, hence we do not present the extended model. The model, posterior checks, and a summary of the parameters are available in the model code in the Appendix.

Prediction of age at PHV: Estimates of the stature velocity curve, maximum velocity, and age at PHV were based on the first and second derivatives of the multilevel polynomial growth model curve based on the population-level parameters [19]. We used the posterior distribution from each group-level parameter of the linear and second-order terms and the population-level third-order term to compute the second derivative equation to predict the age at PHV for each study [3].

**Figure 1:**
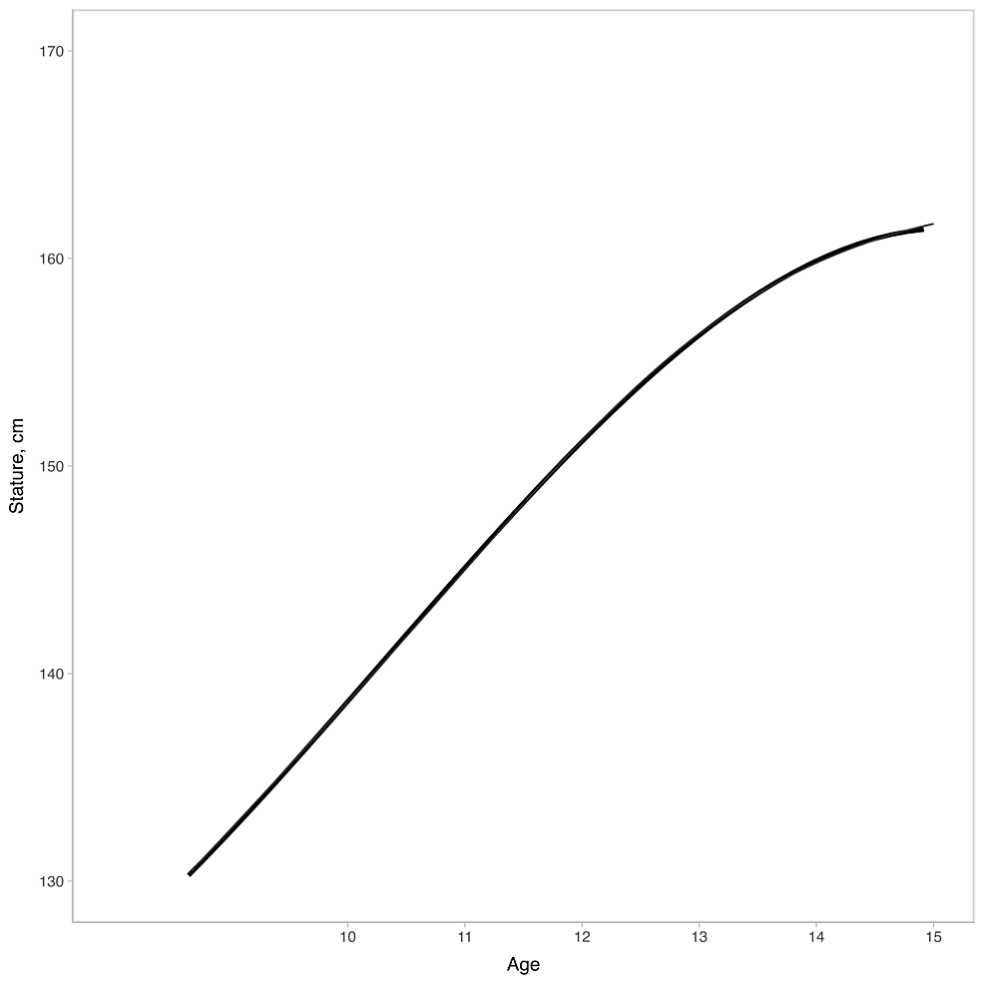
Prior specification model fit against the 50th percentile of the World Health Organization growth charts

Priors and sensitivity analysis: We checked the influence of different prior information on our results by considering non-informative, weakly informative, and informative priors, and comparing predictions. Also, we refitted our model using only studies considered with a low risk of bias. For all prior structures, we specified a weakly regularizing prior for the group-level effects $(\sigma_\alpha, \sigma_{\beta^{age}}, \sigma_{\beta^{age^2}})$, an exponential prior(1). We initially simulated a polynomial growth model based on the World Health Organization growth data [20] to establish the sensitivity of the PHV estimates and to determine informative priors based on reference data. We centered the stature and age at the grand mean to allow stable estimation of the nonlinear parameters in the age range adopted. We then compared our model-generated data against the observed WHO 50th percentile to check our assumption (Figure 1).

To test the sensitivity of our priors choice, we used three priors for the population level effects: non-informative prior,$(\mu,\beta^{age},\beta^{age^2}, \beta^{age^3}) \sim Normal(0,50)$; weakly informative prior, $(\mu,\beta^{age},\beta^{age^2}, \beta^{age^3}) \sim Normal(0,20)$; informative prior,$\mu \sim Normal(2,10), \beta^{age} \sim Normal(7,10), \beta^{age^2} \sim Normal(-0.3,1), \beta^{age^3} \sim Normal(-0.1,1)$.

Risk of bias assessment: We used the adjusted signaling questions for each domain considering specifically our meta-analysis research question and design [3]. Following our previous work with longitudinal meta-analysis (see supplementary material of [3]), we used two approaches to interpret funnel plot asymmetry: the conventional measure, the Egger regression test [15], and an alternative approach using the meta-analysis model regression residuals and the study’s sample size [16]. 

We also refitted our meta-analysis model using only the studies considered with a low risk of bias. The refitted model also showed good predictions compared with the empirical distribution of the stature values extracted from the studies deemed low bias risk. The predicted age at peak height velocity in the refitted model was 13.03 years (90% credible interval 12.67 to 13.3), which was similar to the predictions from our model and with non-informative and informative priors on all available data. Hence, we considered the inclusion of all available data acceptable.

Model comparisons, posterior predictive checks, computation, and transparency: To compare the models with different priors we used leave-one-out cross-validation (LOO-CV) and posterior predictive checks [21,22]. The Bayesian models were fitted using R statistical language [23], “brms” package [24], which call Stan [25]. The model predictions were visualized using the “ggplot2” package [26]. We used the default chain length, i.e. four chains for 2000 iterations with a warm-up length of 1000 iterations. Given the computation time, this was enough length of the chain and warm-up to achieve convergence and obtain a reasonable, effective sample size. A repository containing the data and code necessary to recreate the analyses and figures is available at https://osf.io/znrck/.

Results

**Figure 2:**
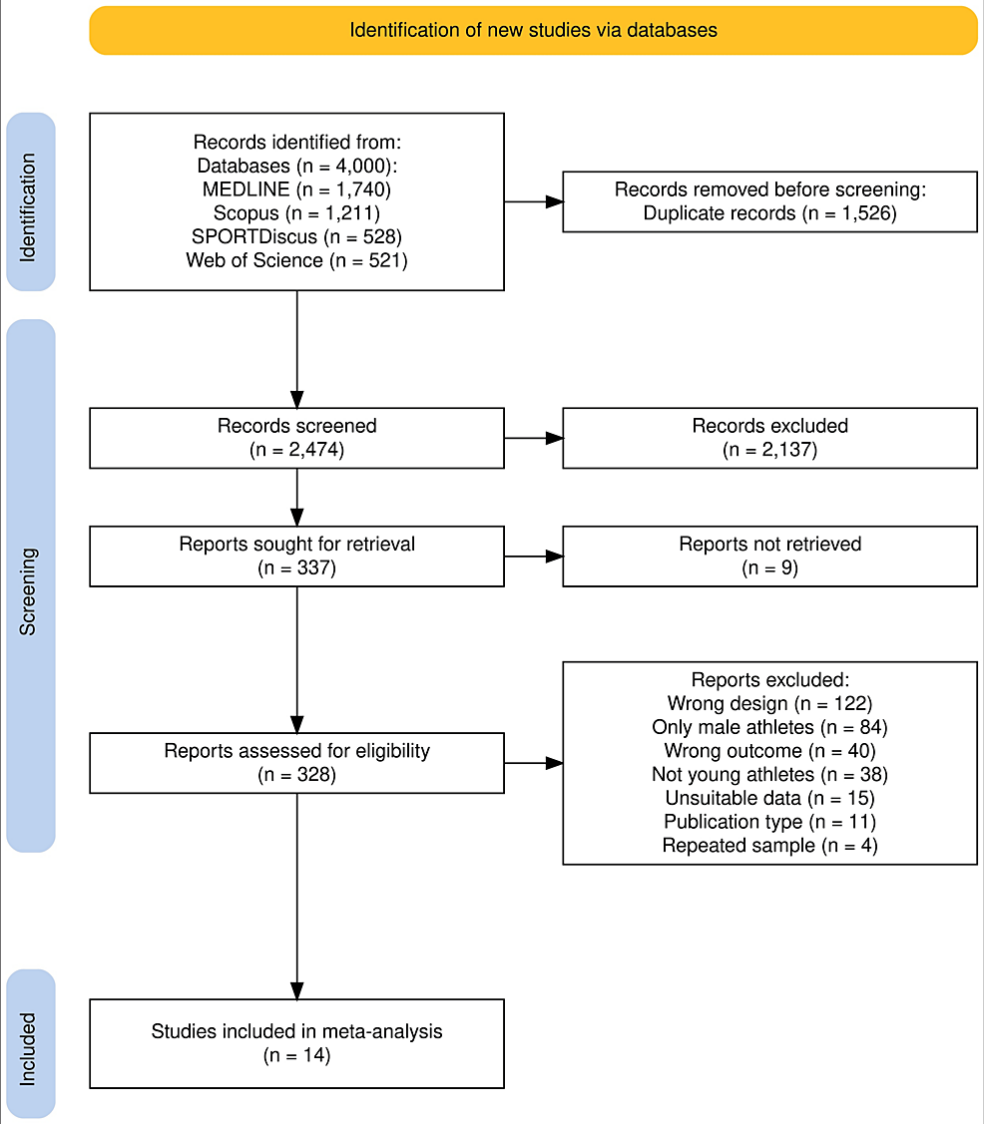
Flowchart including literature search and selection steps following the PRISMA statement

In the systematic review, we initially identified a total of 4,000 articles from electronic databases. During the full-text assessment, the primary reasons for exclusion were inadequate study designs (n = 122) (e.g., cross-sectional studies and those limited to two-point measurements) and studies exclusively involving male athletes (n = 84). It should be noted that articles may have multiple reasons for exclusion, but they were categorized based on the initial reason identified. A total of 14 studies met our eligibility criteria and were included in the meta-analysis (see Figure 2). Among these, four studies contained two or more independent subsamples within the study [27-30], such as “elite” and “sub-elite” athletes, resulting in 21 independent samples considered in our analysis.

Of the 14 studies included in this meta-analysis, five used mixed-longitudinal designs. Of the nine studies that used longitudinal designs, six did not report attrition across the studies' observations. The range of sample sizes varied from seven to 132 athletes. Most of the studies followed the athletes for at least two years (12 out of 14). Only one study was not from the USA or non-European countries [31]. Of the 21 samples, four did not specify the sport or included multiple sports in the same group [30,32-34]. Field Hockey and Tennis had multiple samples but from the same study [28,29]. Multiple samples from different studies were collected for Basketball [31,35], Gymnastics [27,36], and Volleyball [37,38]. Additional study information is presented in Table 1.

**Table 1:** Characteristics of the studies included in the longitudinal meta-analysis ^1 ^Describes the maximum number of subjects throughout the assessment points of the study, considering that most studies vary the sample throughout the study. ^2^ Denominated by the authors as: (a) prepubertal, (b) peripubertal and (c) postpubertal advanced gymnastics, and (d) prepubertal and (e) peripubertal intermediate gymnastics. ^3^ Denominated by the authors as (a) "elite" and (b) "sub-elite" players.

Reference	Sport	Country	Design	Sample^1^	Number of Obs	Interval	Duration
Malina [37]	Volleyball	USA	Longitudinal	19	4	~0.5 year	4 years
Fogelholm et al. [32]	Gymnastics, figure skating, and running	Finland	Longitudinal	28	3	1 year	2 years
Eisenmann and Malina [39]	Running	USA	Longitudinal	16	5	1 year	4 years
Prokopec et al. [38]	Volleyball	Czech Republic	Mixed-longitudinal	58	6	1 year	5 years
Daly et al.^2 ^[27]	Gymnastics	USA	Mixed-longitudinal	15	5	1 year	2 years
Daly et al.^2 ^[27]	Gymnastics	USA	Mixed-longitudinal	30	5	1 year	2 years
Daly et al.^2 ^[27]	Gymnastics	USA	Mixed-longitudinal	17	5	1 year	2 years
Daly et al.^2 ^[27]	Gymnastics	USA	Mixed-longitudinal	23	3	0.5 year	2 years
Daly et al.^2 ^[27]	Gymnastics	USA	Mixed-longitudinal	10	3	0.5 year	2 years
Elferink-Gemser et al.^3 ^[28]	Field hockey	The Netherlands	Mixed-longitudinal	18	3	1 year	2 years
Elferink-Gemser et al.^3 ^[28]	Field hockey	The Netherlands	Mixed-longitudinal	25	3	1 year	2 years
Quatman-Yates et al. [33]	Football and basketball	USA	Longitudinal	39	3	1 year	2 years
Marina and Jemni [36]	Gymnastics	Denmark	Longitudinal	9	6	4 months	20 months
Csajagi et al. [40]	Swimming	Hungary	Longitudinal	7	6	~3 months	1.5 year
Stojmenovic et al. [35]	Basketball	Serbia	Longitudinal	25	6	0.5 year	2.5 years
Holden et al. [34]	High school sports	Ireland	Longitudinal	80	5	0.5 year	2 years
Kramer et al.^3 ^[29]	Tennis	The Netherlands	Mixed-longitudinal	50	9	1 year	8 years
Kramer et al.^3 ^[29]	Tennis	The Netherlands	Mixed-longitudinal	13	9	1 year	8 years
Landgraff et al. [30]	Ski cross-country	Norway	Longitudinal	17	3	1 year	2 years
Landgraff et al. [30]	Team sports	Norway	Longitudinal	9	3	1 year	2 years
Soares et al [31]	Basketball	Brazil	Mixed-longitudinal	40	9	0.5 year	4 years

**Figure 3:**
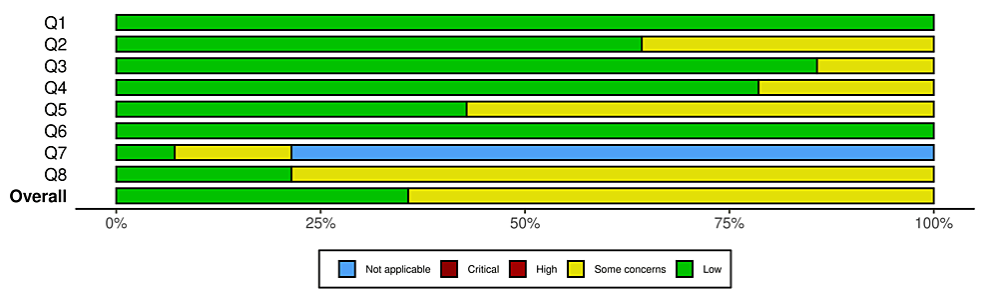
Distribution of degree of judgment by signaling questions in the studies included in the meta-analysis

**Figure 4:**
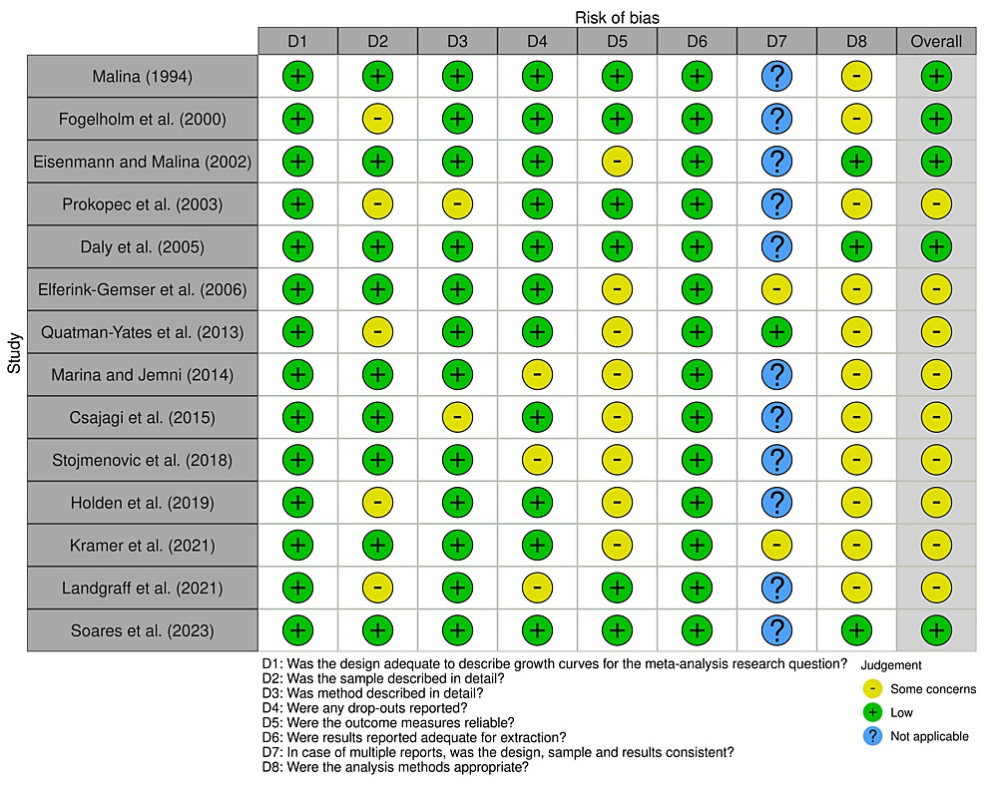
Traffic light plots of the degree of judgment in each signaling question across the studies included in the meta-analysis Malina [37], Fogelholm et al. [32], Eisenmann and Malina [39], Prokopec et al. [38], Daly et al. [27], Elferink-Gemser et al. [28], Quatman-Yates et al. [33], Marina and Jemni [36], Csajagi et al. [40], Stojmenovic et al. [35], Holden et al. [34], Kramer et al. [29], Landgraff et al. [30], Soares et al. [31]

The distribution of the degree of judgment by signaling questions in the studies included in the meta-analysis is presented in Figure 3. The traffic light plot of the degree of judgment in each signaling question across the studies included in the meta-analysis is presented in Figure 4.

**Figure 5:**
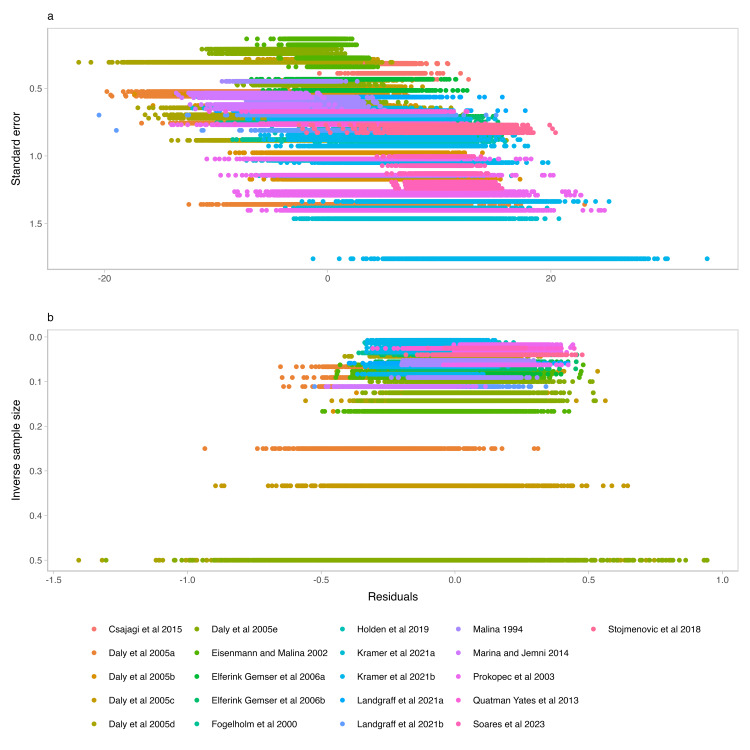
Funnel plots of the meta-analysis of published growth data in young female athletes (a) Distribution of the study's standard error on a reverse scale against the Bayesian meta-analysis model residuals; (b) distribution of the study's inverse sample sizes on a reverse scale against the Bayesian meta-analysis model residuals Stojmenovic et al. [35], Soares et al [31], Quatman-Yates et al. [33], Prokopec et al. [38], Marina and Jemni [36], Malina [37], Landgraff et al. [30], Landgraff et al. [30], Kramer et al. [29], Kramer et al. [29], Holden et al. [34], Fogelholm et al. [32], Elferink-Gemser et al. [28], Elferink-Gemser et al. [28], Eisenmann and Malina [39], Daly et al. [27], Daly et al. [27], Daly et al. [27], Daly et al. [27], Daly et al. [27], Csajagi et al. [40]

The distributions of the study's standard error (Figure 5, panel A) and inverse sample sizes (both on reverse scales) against the Bayesian meta-analysis model residuals (Figure 5, panel B) showed a trend of asymmetry in the funnel plots. We also refitted our meta-analysis model using only the studies considered with a low risk of bias. The refitted model showed good predictions compared with the empirical distribution of the stature values extracted from the studies deemed low bias risk. The predicted age at peak height velocity in the refitted model was 13.03 years (90% credible interval 12.67 to 13.3), which was similar to the predictions from our model and with non-informative and informative priors on all available data. Hence, we considered the inclusion of all available data acceptable. Based on our quality criteria, the overall quality of the included studies was deemed acceptable. However, certain limitations in the reports led us to adopt a more conservative interpretation of our meta-analysis model predictions.

**Figure 6:**
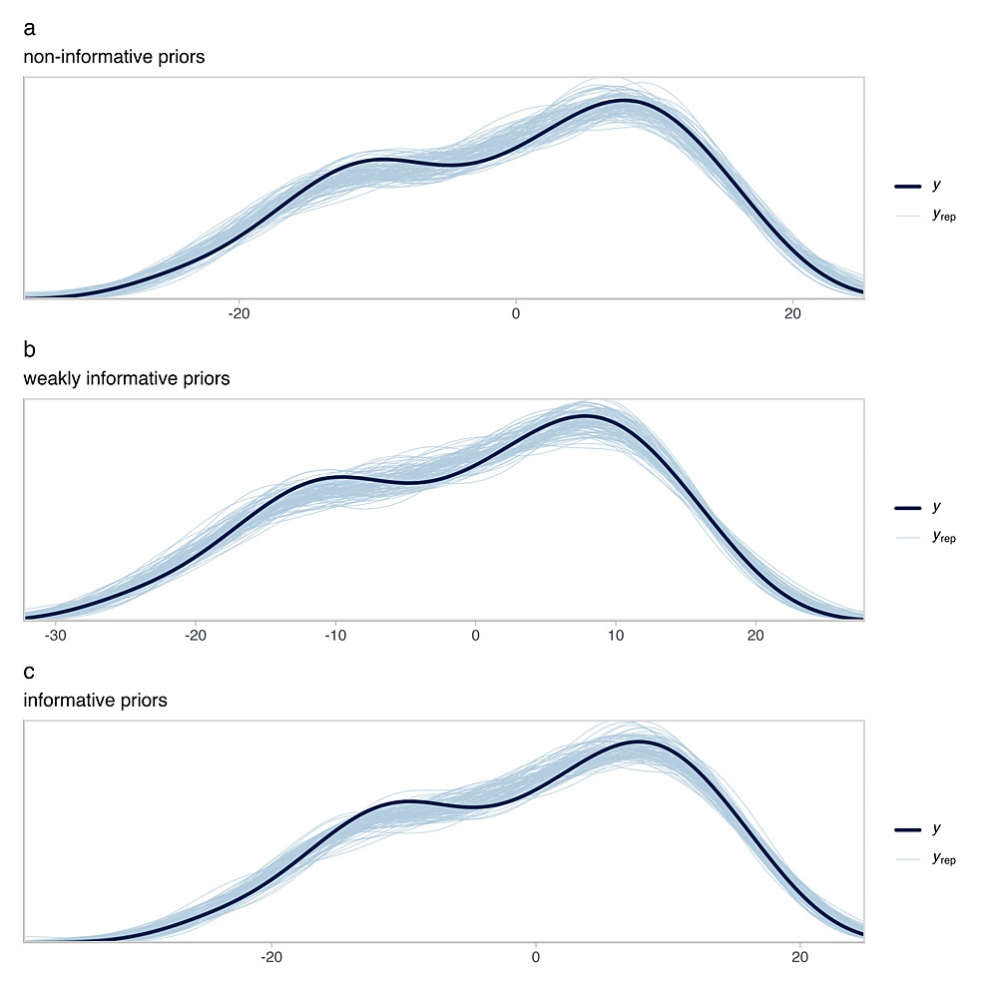
Distributions of replicated data sets drawn from the posterior predictive distribution in our models with different priors structures compared with the empirical distribution of the stature values extracted from the literature. (a) Posterior predictive checks for the model with non-informative priors; (b) posterior predictive checks for the model with weakly informative priors; (c) posterior predictive checks for the model with informative priors

The predictive performance was similar across the models with the three different prior structures. Also, the posterior predictive checks showed that the distribution function of the data could reasonably fall within the simulated data, suggesting that the three models could have reasonably generated the data (Figure 6).

**Figure 7:**
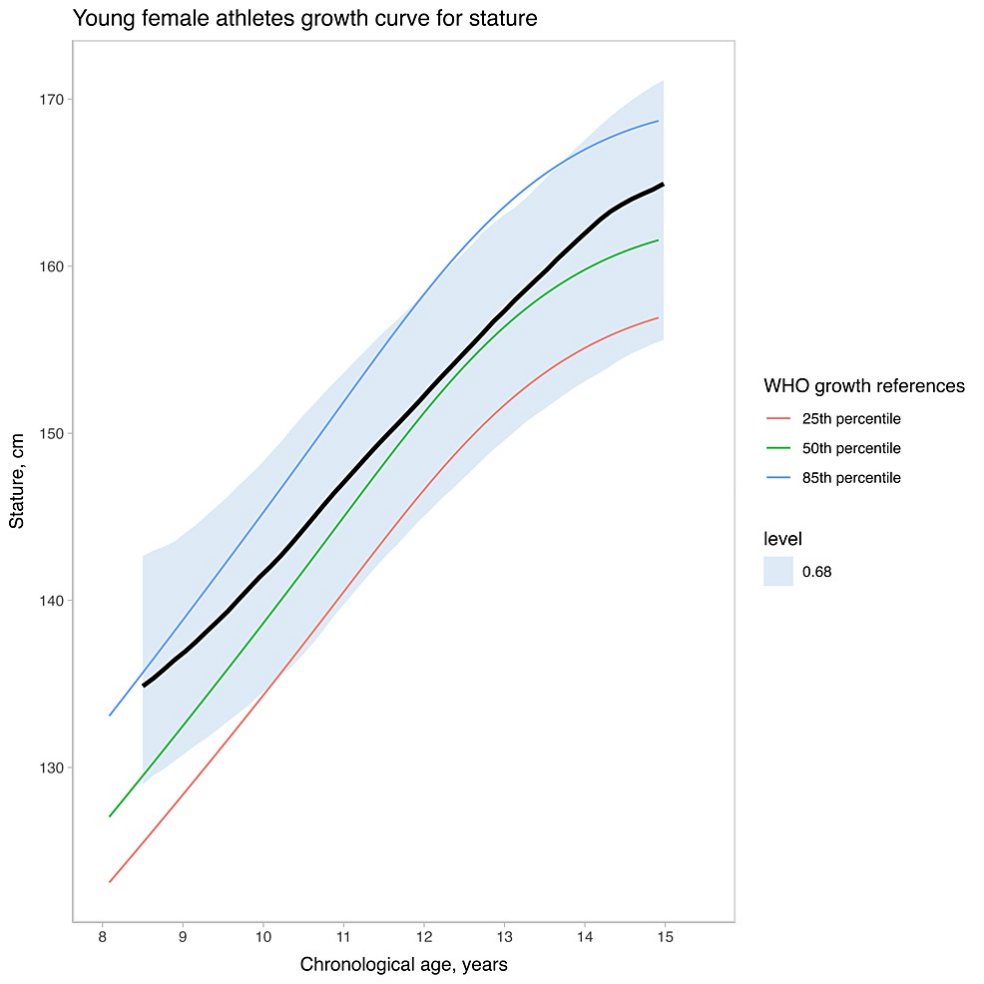
Growth curve of young female athletes (stature by chronological age) contrasted against the World Health Organization growth references. Error bars indicate the 68% credible interval

**Figure 8:**
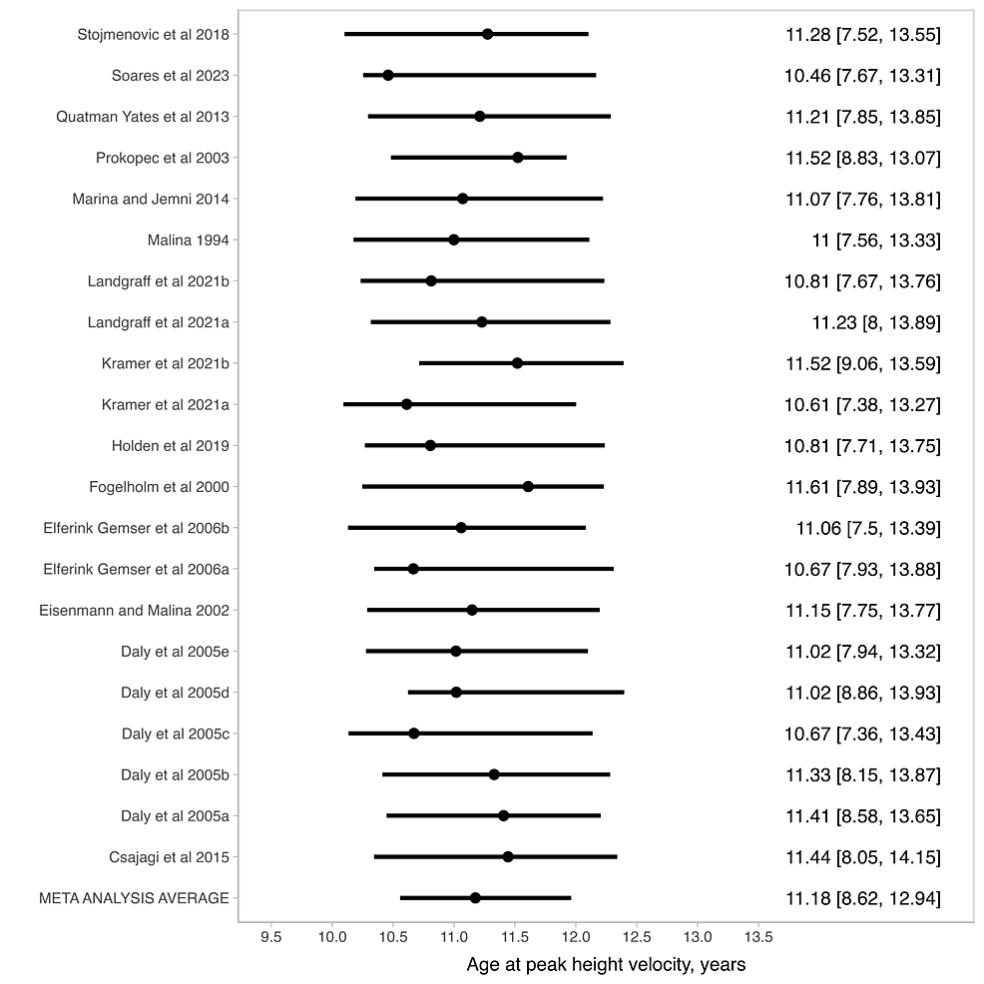
Estimates of age at peak height velocity of young female athletes by study included in the longitudinal meta-analysis. Error bars indicate the 68% credible interval Stojmenovic et al. [35], Soares et al. [31], Quatman-Yates et al. [33], Prokopec et al. [38], Marina and Jemni [36], Malina [37], Landgraff et al. [30], Landgraff et al. [30], Kramer et al. [29], Kramer et al. [29], Holden et al. [34], Fogelholm et al. [32], Elferink-Gemser et al. [28],  Elferink-Gemser et al. [28], Eisenmann and Malina [39], Daly et al. [27], Daly et al. [27], Daly et al. [27], Daly et al. [27], Daly et al. [27], Csajagi et al. [40]

The meta-analysis growth curve for stature is displayed in Figure 7. We plotted the estimated stature against the World Health Organization (WHO) reference growth curves [20]. Considering the study data and weakly informative priors, the average age at PHV for the total sample of studies with young female athletes was 11.18 years (90% credible interval (CI): 8.62; 12.94). For informative priors, the estimated age at PHV was 11.20 (90% CI: 8.94; 12.58), while for non-informative priors the estimated age at PHV was 11.36 (90% CI: 8.72; 12.87). There was substantial variation across studies (see Figure 8).

Discussion

In our current meta-analysis, we evaluated growth curves and determined the age at PHV in young female athletes from a Bayesian perspective. To our knowledge, this is the first comprehensive analysis of growth patterns and prediction of age at PHV specifically in female athletes. Conditional on the data and models, the predicted mean age at PHV for female athletes was 11.18 years (90% CI: 8.62; 12.94). When studies were aggregated by sport in the models, the models could not capture sport-specific growth curves for stature and estimate a corresponding age at PHV.

Taking into account potential differences in estimation methods, the predictions and uncertainty estimates of our study are consistent with findings from non-athlete populations. A review of growth studies conducted in North America and Europe found that girls typically experience their PHV between 11.4 and 12.2 years [41]. Based on a meta-analysis of the age at PHV estimates from the growth studies [41], the estimated age at PHV was 11.9 years (90% CI: 11.8, 12.0) [42]. On the other hand, in our prior prediction checks, we estimated an age at PHV of 10.3 years for the 50th percentile estimates of the WHO reference growth data for girls [20]. Discrepancies in estimates from growth studies may be explained by differences in methods used to derive age at PHV and data structures. Comparisons of methods for estimating age at PHV have found differences in estimates whether using simulated data [17,43,44] or real growth data [41]. In addition, WHO growth standards have been established using extensive and geographically diverse data, combining both longitudinal follow-up and cross-sectional data [45]. In contrast, growth studies rely primarily on longitudinal follow-up with limited available data, often requiring complete data for each individual and from specific geographic contexts [1].

Fitting models using a multilevel framework naturally and explicitly model data structure complexities, such as imbalanced samples [46]. Longitudinal meta-analysis exemplifies complex data structures. Additionally, by fitting our models using a fully Bayesian framework, we incorporated available knowledge to regularize the models’ shape and magnitude, potentially overcoming the limits of multilevel polynomial growth [3]. Bayesian modeling allows us to combine prior knowledge via the specification of prior distributions in the model with the available data to arrive at a posterior distribution that places higher credibility on parameters more consistent with the data [47]. We tested the effect of prior information on the interpretation and sensitivity of our estimates by comparing model fits with non-informative, weakly regularized, and informative (based on WHO data) model fits. Our findings indicate consistent posterior estimates, leading us to believe that the studies included in the meta-analysis had sufficient information to describe the growth curves of young female athletes.

Growth studies have been well-established in the scientific community since 1900 [48]. However, the first study involving young female athletes was not published until 1994 by Malina and colleagues [37], seven years after the first publication focusing on male athletes [49]. Despite this, the production of longitudinal growth studies in female athletes has been irregular over time. Only since 2013 has there been a modest increase in the frequency of published studies. Until 2013, only six studies had been published. While the production of longitudinal studies on male athletes is less abundant, with a total of only 31 studies identified in our previous review [3], the number of studies on female athletes is less than half that amount. Our review supports the contention that females are underrepresented in sports medicine-related studies [5, 50]. Furthermore, the availability of limited specific data poses challenges in establishing guidelines for the training and performance of female athletes [6-8].

A recent increase in the number of publications could be explained by the growing prominence and performance of women in major sporting events [4]. Despite progress, there is still much room for advancement in the field of sports science. However, it is necessary to design longitudinal studies to monitor the growth of female athletes [51]. It is becoming increasingly important to adopt good research practices and address issues such as low research transparency, wasted data, and inaccessibility [51-53]. In addition, more literature on female athletes has emerged. However, some of these studies were not included in the meta-analysis due to unavailable data, restricted access to research, and unreliable or incomplete data. This illustrates a clear and unfortunate example of wasted data.

Furthermore, the meta-analysis reveals that there is potential for improvement in the studies examined. In numerous cases, the experimental methodology lacks clarity, with no accompanying materials aside from the journal article, such as study registration or protocol. Athletes’ descriptions often lack important details such as the sport context, athlete characteristics, and, in the case of mixed longitudinal designs, comprehensive information on participants' entry and dropout. Even pure longitudinal studies often fail to report the total number of dropouts throughout the study. Supplementary materials containing more detailed data and open-access datasets are rarely provided with journal articles. Improving these inadequacies would notably increase the transparency, reproducibility, and overall quality of research in our field.

Given the diverse ethnic variations across and within nations and continents, it is important to conduct global studies to better understand the growth and maturation of athletes. This approach ensures a comprehensive understanding that takes into account the unique characteristics of each region. Our analysis revealed minimal variability, as over 90% of the studies come from either the United States [27,33,37,39] or European nations: Czech Republic [38], Denmark [36], Finland [32], Hungary [40], Ireland [34], Norway [30], Serbia [35], and the Netherlands [28, 29]. Only one study, conducted in Brazil [31], was not performed in this group of countries. Notably, there is a complete absence of longitudinal data for athletes from entire continents like Africa and Asia. Furthermore, even within Europe, the most knowledge-producing continent, data from countries with well-established women’s sports systems, such as England and Spain, are lacking. This is an important limitation that highlights the need for more comprehensive and globally representative research in this field.

Limited information availability results in singular studies in field hockey [28], swimming [40], tennis [29], ski cross-country [30], and running [39]. However, data for other sports, notably football, are absent despite its global popularity. Even in the study by Quatman-Yates et al. [33], which included football athletes, data were combined with basketball, hindering specific sports insights. Some studies lack sufficient sports context, complicating sample identification. Only basketball [31,35], gymnastics [27,36], and volleyball [37,38] have multiple studies, enabling more comprehensive information extraction.

Caution should be exercised when making generalizations based on our predictions. Our analysis only considers limited longitudinal data. Multilevel polynomial growth models are not the only method. It is important to note that there are other modeling options available within a multilevel framework to model pubertal growth for stature, such as multilevel models with natural cubic splines (SuperImposition by Translation And Rotation, SITAR) [54]. However, SITAR may have limitations when data quality is not optimal (such as with meta-analysis), which could result in unreasonable estimations, and model convergence is often difficult to achieve [17,44].

## Conclusions

In summary, this study provides a longitudinal meta-analytical review of pubertal growth and derives the age at PHV in young female athletes. Thus, we present a replicable template that can be reproduced and hopefully with more data to improve the confidence in the predictions. Our analysis suggests that the age at PHV in the longitudinal data of young athletes in the literature occurs at similar ages to those in the general pediatric population. However, the data pool was limited across a range of studies, with variations by sport and geographical distribution. In this study, we adhere to the calls for research transparency. Our statistical framework is robust and employs explicit model criticism, including posterior predictive checking. Anyone interested in our data can easily modify the framework.

## Appendices

The search strategy employed utilized the search terms outlined in Table 2 across multiple databases, including MEDLINE (PubMed), Web of Science, SPORTDiscus (EBSCOhost), and SCOPUS. The search terms were systematically organized into blocks corresponding to the PICOS framework, covering Patients/Population (two blocks), Intervention/Sports, Outcomes, and Study Type. Each database received a comprehensive set of search terms tailored to its specificity. Following individual searches within each block, a combined search was conducted to ensure a thorough exploration of the literature. The first search was performed on March 13, 2020, and updates were conducted by rerunning searches.

**Table 2:** Terms used during electronic databases search

Block 1	Patients/Population AND	young OR youth OR adolescent OR teen OR youths OR adolescents OR teens OR child OR children OR teenagers OR teenager
Block 2	Patients/ Population AND	players OR player OR athletes OR athlete
Block 3	Intervention/ Sports AND	sports OR sport OR soccer OR football OR basketball OR volleyball OR handball OR hockey OR cricket OR baseball OR rugby OR lacrosse OR softball OR netball OR bicycling OR boxing OR golf OR gymnastics OR “martial arts” OR tennis OR running OR skating OR skiing OR “track and field” OR swimming OR mountaineering OR “racquet sports” OR “water sports” OR walking OR “weight lifting” OR wrestling OR athletics
Block 4	Outcomes AND	sprint OR jump OR “aerobic endurance” OR “physical endurance” OR strength OR power OR agility OR “medicine ball throw” OR “physical performance” OR “functional performance” OR “physical fitness” OR “functional capacities” OR “functional capacity” OR “physiological responses” OR “physiological response” OR “physiological adaptations” OR “physiological adaptation” OR height OR stature OR “body mass” OR “body weight” OR vo2 OR “peak height velocity” OR PHV OR maturation OR “short-term power output”
Block 5	Study type	longitudinal OR “repeated measures” OR “repeated measure”

MEDLINE (via PubMed, 1946 to present)

Date of last search: 2023-08-23.

Field codes: None.

Limits and restrictions: Language = “English”.

Search filters: None.

Total number of hits: 1,740 hits.

Full search strategy: (young OR youth OR adolescent OR teen OR youths OR adolescents OR teens OR child OR children OR teenagers OR teenager) AND (players OR player OR athletes OR athlete) AND (sports OR sport OR soccer OR football OR basketball OR volleyball OR handball OR hockey OR cricket OR baseball OR rugby OR lacrosse OR softball OR netball OR bicycling OR boxing OR golf OR gymnastics OR “martial arts” OR tennis OR running OR skating OR skiing OR “track and field” OR swimming OR mountaineering OR “racquet sports” OR “water sports” OR walking OR “weight lifting” OR wrestling OR athletics) AND (sprint OR jump OR “aerobic endurance” OR “physical endurance” OR strength OR power OR agility OR “medicine ball throw” OR “physical performance” OR “functional performance” OR “physical fitness” OR “functional capacities” OR “functional capacity” OR “physiological responses” OR “physiological response” OR “physiological adaptations” OR “physiological adaptation” OR height OR stature OR “body mass” OR “body weight” OR vo2 OR “peak height velocity” OR PHV OR maturation OR “short-term power output”) AND (longitudinal OR “repeated measures” OR “repeated measure”)

Web of Science (core collection, 1945 to present)

Date of last search: 2023-08-23.

Field codes: Topic (“TS”).

Limits and restrictions: Language = “English”; Document Type = “Article”, “Book Chapter”.

Search filters: None.

Total number of hits: 521 hits.

Full search strategy: (TS=(young OR youth OR adolescent OR teen OR youths OR adolescents OR teens OR child OR children OR teenagers OR teenager)) AND (TS=(players OR player OR athletes OR athlete)) AND (TS=(sports OR sport OR soccer OR football OR basketball OR volleyball OR handball OR hockey OR cricket OR baseball OR rugby OR lacrosse OR softball OR netball OR bicycling OR boxing OR golf OR gymnastics OR “martial arts” OR tennis OR running OR skating OR skiing OR “track and field” OR swimming OR mountaineering OR “racquet sports” OR “water sports” OR walking OR “weight lifting” OR wrestling OR athletics)) AND (TS=(sprint OR jump OR “aerobic endurance” OR “physical endurance” OR strength OR power OR agility OR “medicine ball throw” OR “physical performance” OR “functional performance” OR “physical fitness” OR “functional capacities” OR “functional capacity” OR “physiological responses” OR “physiological response” OR “physiological adaptations” OR “physiological adaptation” OR height OR stature OR “body mass” OR “body weight” OR vo2 OR “peak height velocity” OR PHV OR maturation OR “short-term power output”)) AND (TS=(height OR stature OR “body mass” OR “body weight” OR “peak height velocity” OR PHV OR maturation OR anthropometry OR anthropometric OR anthropometrics)) AND (TS=(longitudinal OR “repeated measures” OR “repeated measure”)) AND (DT=(Article OR Book Chapter)) AND (LA=(English))

SCOPUS

Date of last search: 2023-08-23.

Field codes: Article title, abstract, keywords(TITLE-ABS-KEY) 

Limits and restrictions: Language = “English”; Document Type = “Article”; Source Type = “Journal Article”.

Search filters: None.

Total number of hits: 1,211 hits.

Full search strategy: (((TITLE-ABS-KEY (young OR youth OR adolescent OR teen OR youths OR adolescents OR teens OR child OR children OR teenagers OR teenager))) AND ((TITLE-ABS-KEY (players OR player OR athletes OR athlete))) AND ((TITLE-ABS-KEY (sports OR sport OR soccer OR football OR basketball OR volleyball OR handball OR hockey OR cricket OR baseball OR rugby OR lacrosse OR softball OR netball OR bicycling OR boxing OR golf OR gymnastics OR “martial arts” OR tennis OR running OR skating OR skiing OR “track and field” OR swimming OR mountaineering OR “racquet sports” OR “water sports” OR walking OR “weight lifting” OR wrestling OR athletics))) AND ((TITLE-ABS-KEY (sprint OR jump OR “aerobic endurance” OR “physical endurance” OR strength OR power OR agility OR “medicine ball throw” OR “physical performance” OR “functional performance” OR “physical fitness” OR “functional capacities” OR “functional capacity” OR “physiological responses” OR “physiological response” OR “physiological adaptations” OR “physiological adaptation” OR height OR stature OR “body mass” OR “body weight” OR vo2 OR “peak height velocity” OR phv OR maturation OR “short-term power output”))) AND ((TITLE-ABS-KEY (longitudinal OR “repeated measures” OR “repeated measure”)))) AND (LIMIT-TO (SRCTYPE , “j”)) AND (LIMIT-TO (DOCTYPE , “ar”)) AND (LIMIT-TO (LANGUAGE , “English”)

SPORTDiscus (via EBSCOhost)

Date of last search: 2022-08-31*.

Field codes: None. 

Limits and restrictions: Language = “English”; Document Type = “Article”; Publication Type = “Academic Journal”.

Search filters: None.

Total number of hits: 528 hits.

Full search strategy: (young OR youth OR adolescent OR teen OR youths OR adolescents OR teens OR child OR children OR teenagers OR teenager) AND (players OR player OR athletes OR athlete) AND (sports OR sport OR soccer OR football OR basketball OR volleyball OR handball OR hockey OR cricket OR baseball OR rugby OR lacrosse OR softball OR netball OR bicycling OR boxing OR golf OR gymnastics OR “martial arts” OR tennis OR running OR skating OR skiing OR “track and field” OR swimming OR mountaineering OR “racquet sports” OR “water sports” OR walking OR “weight lifting” OR wrestling OR athletics) AND (sprint OR jump OR “aerobic endurance” OR “physical endurance” OR strength OR power OR agility OR “medicine ball throw” OR “physical performance” OR “functional performance” OR “physical fitness” OR “functional capacities” OR “functional capacity” OR “physiological responses” OR “physiological response” OR “physiological adaptations” OR “physiological adaptation” OR height OR stature OR “body mass” OR “body weight” OR vo2 OR “peak height velocity” OR PHV OR maturation OR “short-term power output”) AND (longitudinal OR “repeated measures” OR “repeated measure”) AND (Language=English) AND (Document Type=Article) AND (Publication Type=Academic Journal)
